# Exposure to diesel exhaust induces changes in EEG in human volunteers

**DOI:** 10.1186/1743-8977-5-4

**Published:** 2008-03-11

**Authors:** Björn Crüts, Ludo van Etten, Håkan Törnqvist, Anders Blomberg, Thomas Sandström, Nicholas L Mills, Paul JA Borm

**Affiliations:** 1 Centre of Expertise in Life Sciences, Zuyd University, Heerlen, The Netherlands; 2 Department of Respiratory Medicine and Allergy, University of Umeå, Sweden; 3 Centre for Cardiovascular Sciences, The University of Edinburgh, UK

## Abstract

**Background:**

Ambient particulate matter and nanoparticles have been shown to translocate to the brain, and potentially influence the central nervous system. No data are available whether this may lead to functional changes in the brain.

**Methods:**

We exposed 10 human volunteers to dilute diesel exhaust (DE, 300 μg/m^3^) as a model for ambient PM exposure and filtered air for one hour using a double blind randomized crossover design. Brain activity was monitored during and for one hour following each exposure using quantitative electroencephalography (QEEG) at 8 different sites on the scalp. The frequency spectrum of the EEG signals was used to calculate the median power frequency (MPF) and specific frequency bands of the QEEG.

**Results:**

Our data demonstrate a significant increase in MPF in response to DE in the frontal cortex within 30 min into exposure. The increase in MPF is primarily caused by an increase in fast wave activity (β2) and continues to rise during the 1 hour post-exposure interval.

**Conclusion:**

This study is the first to show a functional effect of DE exposure in the human brain, indicating a general cortical stress response. Further studies are required to determine whether this effect is mediated by the nanoparticles in DE and to define the precise pathways involved.

## Introduction

Several epidemiological studies have identified diesel exhaust as an important component in determining the adverse health effects of particulate matter (PM) air pollution [1,2]. The molecular toxicity of diesel exhaust [3], is suggested to include oxidative stress-mediated inflammation, through particle surface, polycyclic aromatic hydrocarbons and redox active metals. Inflammation is considered to be central to both the pulmonary and systemic adverse health effects of diesel through environmental PM exposure [4]. Over the past decades several, several studies have suggested that inhaled nanoparticles are able to translocate to the brain via the olfactory nerves [5,6], where they have been associated with inflammatory changes at sites of deposition [7,8]. Passage to the brain is of particular concern since nanoparticles are potent inducers of oxidative stress [4,9] and the brain is very sensitive to damage caused by oxidative stress [10]. Oxidative stress has been implicated in the pathogenesis of neurodegenerative diseases such as Parkinson's and Alzheimer's disease and it is conceivable that the long-term effects of PM exposure might include a decrease in cognitive function [11]. Exposure to PM in an experimental mouse model resulted in widespread activation of pro-inflammatory cytokines in the brain [7].

First epidemiological evidence for a functional effect of PM on brain function was presented in recent study that suggests an association between black carbon levels in the environment and cognitive development of children[12]. As a first experimental step to investigate functional effects of PM and its ultrafine fraction, we studied the short-term changes in brain activity induced by exposure to diesel exhaust. We exposed human volunteers for one hour to diesel exhaust (DE, 300 μg/m3) and filtered air and followed their brain activity using quantitative electroencephalography (QEEG) atdifferent sites of the scalp during and after one hour after exposure. The exposure protocol reflects a peak exposure that may occur in environmental and occupational exposure to diesel exhaust.

## Methods

Ten subjects (all male, mean age: 26 years, range: 18–39 years; free of neurological or psychopathological impairments) were exposed to diesel exhaust and filtered air during one hour (sham condition) in a blinded randomized crossover design, separated by a period of two to four days. Prior to the start of each exposure, resting brain activity was measured using QEEG outside the exposure chamber during 3-minute eyes open (Table 1) and 3-minute eyes closed periods. After the 1 hour exposure, EEG measurements were continued for another hour. Participants gave written informed consent andthe study was approved by the Ethics Committee of the University Hospital of Umeå. Diesel exhaust was produced by a Volvo Diesel engine (Volvo TD45, 4.5 L, 4 cylinders, 680 rpm) as described previously [13], leading to 1.2 × 10^6 ^suspended particles/cm^3 ^(300 μg/m^3^) and gaseous pollutant levels of nitrogen dioxide (NO_2_, 1.6 ppm, Nitrogen oxide (NO), 4.5 ppm, carbon monoxide (CO),7.5 ppm and total hydrocarbons of 4.3 ppm.

**Table 1:** Individual absolute values of the median power frequency (MPF) at frontal locations (Fp1) and central location (C3) at pre- and post-exposure measurements (3 min each).

Nr	**MPF at Fp1 (Hz)**				**MPF at C3 (Hz)**			
	Sham		**Diesel**		Sham		**Diesel**	

	pre	post	**pre**	**post**	pre	post	**pre**	**post**

1	5.7	7.6	**6.4**	**8.0**	6.5	8.7	**6.9**	**9.4**
2	7.1	8.3	**8.7**	**12.6**	7.8	6.1	**9.5**	**11.2**
3	7.8	8.5	**7.4**	**8.4**	9.7	8.4	**6.7**	**10.2**
4	8.3	7.7	**8.7**	**9**	11.5	9.9	**9**	**9.6**
5	9.2	9.1	**9.8**	**8.4**	10.5	12.3	**9.7**	**9.2**
6	7.1	10.7	**8.9**	**11**	11	11	**9.0**	**11.9**
7	15.3	12.7	**9.3**	**14.4**	15.1	13.6	**7.9**	**9.1**
8	10.2	11	**7.4**	**11.3**	7.6	9.1	**8.3**	**9.3**
9	7.5	7.3	**6.6**	**7.1**	7	6.3	**7.2**	**8.0**
10	6.7	7	**8.9**	**11.2**	11.2	12.3	**7.4**	**9.1**
***Mean***	***8.5***	***9.0***	***8.2***	***10.1***^a^	***9.9***	***9.8***	***8.2***	***9.7***^a^
***SD***	***2.7***	***1.9***	***1.2***	***2.3***	***2.6***	***2.6***	***1.1***	***1.1***

Measurements were done before entering the exposure chamber and after completion of post-exposure interval, in exposure conditions sham and diesel.

a) Significantly different from pre-exposure, P < 0.02, Wilcoxon paired test

During the EEG measurement, subjects rested in silence and performed no exercise to avoid interference on the QEEG measurement. Subjects sat in an upright position during the exposures, and were allowed to read a book to avoid boredom. EEG was continuously recorded from 8 electrode sites on the scalp according to the international 10–20 system: frontal pole (Fp1, Fp2), frontal (F3, F4), central (C3, C4) and parietal (P3, P4) at a sample rate of 500 Hz. The frequency content of the filtered EEG signals was calculated using Fast Fourier Transform (FFT) of 15 seconds intervals. From these intervals the Median Power Frequency (MPF) and spectral bands of the EEG were calculated. A second more detailed analysis divided the power spectrum into distinct frequency bands, including delta (1–3,5 Hz), theta (3,5–7,5 Hz), alpha (7,5–12 Hz), beta1 (12–20 Hz) and beta2 (20–32 Hz) bands. Changes in MPF and spectral bands during and after exposure are first presented as time series. From these time series, the mean MPF and spectral bands per hour and amplitudes of the first and last 5 minutes per exposure or post-exposure interval were calculated. Significance levels of differences (P < 0.05) between the first and last 5 minutes were compared between diesel and sham conditions by applying paired Wilcoxon tests to group results.

## Results

### Changes in Median Power Frequency (MPF)

**Figure 1:**
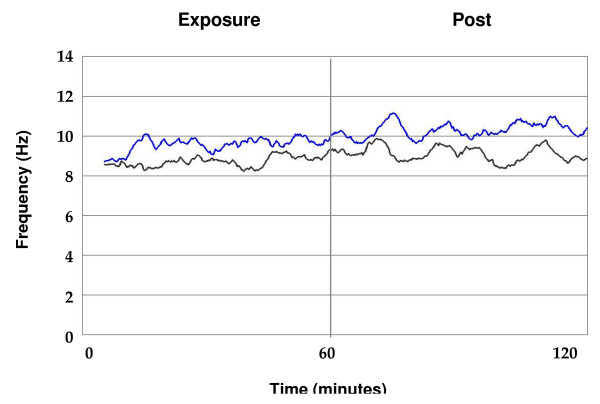
**Time series of MPF at the left frontal cortex (F3)**. Changes in MPF are represented as a function of time in a superposed epoch analysis graph for all subjects combined. Values for MPF are visualized for both diesel (blue line) and sham condition (black line) during the exposure and post exposure period. Both lines represent 5 minute moving averages of the original signal. MPF values during the diesel exposure increase and remain elevated during the post exposure period condition.

**Figure 2:**
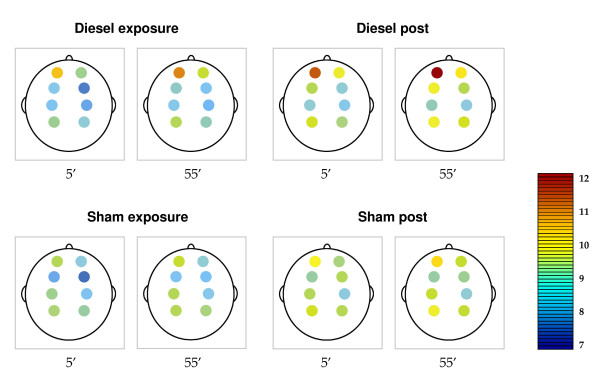
**MPF values per electrode localisation**. The figures represent the 8 electrode localizations, from the frontal areas at the top (Fp1, Fp2) to the parietal areas at the bottom (P3, P4). Amplitudes of MPF are indicated by colors, ranging from deep blue (7 Hz) to red (12 Hz). MPF amplitudes are presented as mean values of the first and last 5 minutes of the exposure hour and post exposure hour. The highest values for MPF during diesel exposure are observed at the frontal polar electrode sites (Fp1, Fp2). At these sites MPF increases significantly during diesel exposure compared to sham exposure. Following the diesel exposure MPF continued to increase resulting in significant differences at the frontal polar and the frontal sites (Fp1, Fp2, F3, F4) compared to the post-sham exposure period.

Baseline measurements of MPF in the exposure chamber did not differ from pre-exposure measurements obtained outside the exposure chamber (data not shown). This shows that there is no acute effect of being in the exposure chamber, such as the presence of a pungent smell remaining after diesel sessions. There were also no significant differences at any time in MPF between subjects during the first 5 minutes of sham or diesel exposure. From 30 minutes into exposure we observed a slow increase in MPF of all subjects within the exposure chamber which was stronger in diesel exhaust exposure as compared to filtered air (Fig. 1). The effect on MPF was first observed and most pronounced at the frontal electrode sites Fp1, Fp2, F3, and F4 (Fig. 2) and the average MPF at Fp1 and Fp2 was significantly different at the end of the diesel exhaust exposure compared to the start of exposure and to the sham conditions (P < 0.05 for both, Table 1). Interestingly the effect further increased during the diesel post-exposure period, when subjects were removed from the exposure chamber. This led to a significant difference between the 3 minute pre- and post-exposure measurements of MPF atthe start and end of the post-exposure period both frontal (Fp1) and at the central (C3) location (Table 1). In addition the change over the exposure intervals, calculated from the difference between the first and last 5 minutes into the chamber, was significantly different between sham and diesel exposure (Table 2). This again indicated that tasks or factors present in the exposure chamber are unlikely to play a role in this effect on EEG. In addition, during post-exposure the effect on MPF spread in the other frontal F3 and F4 electrode sites leading to significant differences betweendiesel and sham (Table 2). Although slower changes in MPF wereobserved at the central (C3, C4) and parietal (P3, P4) electrode sites during the diesel (Fig 2), at the end of exposure the mean MPF at post-exposure measurement was also significantly different from pre-exposure (Table 1).

**Table 2:** Mean absolute change in median power frequency (MPF) at different locations during different exposure conditions.

**Condition**	**Electrode locations**							
	**Fp1**	**Fp2**	**F3**	**F4**	**C3**	**C4**	**P3**	**P4**
	
**ΔDiesel**	0,41*	0,34*	0,40	0,54	0,12	0,18	0,21	0,24
**ΔSham**	0,10	0,12	0,34	0,50	0,23	0,13	0,17	0,25

**ΔDiesel post**	0,46*	0,43*	0,41*	0,60*	0,23	0,20	0,23	0,30
**ΔSham post**	0,21	0,24	0,09	-0,20	0,18	0,15	0,21	0,19

Mean changes in the median power frequency between the first and last 5 minutes of all conditions are shown per electrode localization. * Significant differences between diesel and sham exposures, Wilcoxon paired test (P < 0.05).

### Analysis of EEG frequency bands

**Figure 3:**
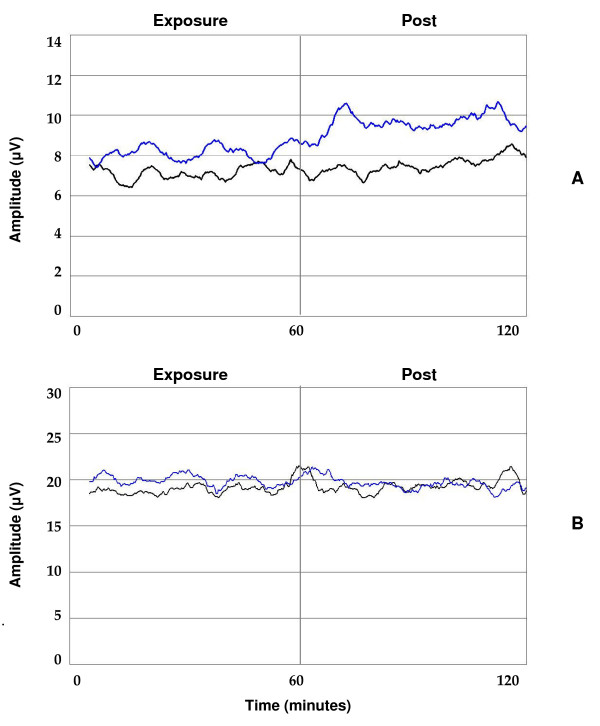
**Absolute beta2 amplitudes (A) and theta amplitudes (B) at the left frontal cortex (F3)**. Changes are represented as a function of time in a superposed epoch analysis graph for all subjects combined. Absolute power per frequency band is visualized for diesel (blue line) and sham condition (black line), combining the one hour exposure condition and subsequent post condition in one graph. Both lines represent a 5 minute moving average of the original signal. Beta2 (A) but not theta-amplitudes (B) are elevated during diesel exposure compared to sham exposure.

Subsequently we extended the data-analysis to determine the effects of diesel exhaust on specific frequency bands from the EEG signal. The increased MPF at frontal electrode sites (Fp1, Fp2, F3, F4), is largely explained by an effect on fast wave activity (20–32 Hz, also denominated as beta2 activity). During diesel exposure, β2 activity increased significantly compared to sham exposure. A marked increase in the β2 signal was observed at the end of the diesel exposure interval in 7 out of 10 subjects (Fig. 3). The resulting change remained throughout the entire post exposure period. However, no such change in β2 activity was observed at sham exposure (Fig. 3). Similar changes in fast wave activity were not observed at central and parietal electrode sites. Time variations of all frequency bands were analyzed using short-term Fourier-transformation, which revealed large variations in fast wave activity, with the largest variations over time in β2 values (data not shown). β1-activity (15–20 Hz) also increased during diesel exposure at the frontal cortex, but this rise did not reach statistical significance (data not shown). Delta, theta and alpha activity showed no significant alterations during the diesel and post exposure periods compared to sham conditions (Fig. 3).

## Discussion

This is the first study to demonstrate functional changes in brain activity as a result of exposure to diesel exhaust (DE) in human subjects. Our data demonstrate a delayed response to DE in the frontal cortex, characterized by an increase of median power frequency (MPF) and fast wave activity (21–32 Hz) in the EEG. These findings suggest an increased activity of the left frontal cortex during and after DE, but may be mediated by a number of different pathways.

As the first study of its kind interpretation of these findings are limited by a number of factors. Perhaps the most important limitation of our study is the fact that exposure to diesel exhaust is a mixture of combustion derived nanoparticles (CDNP) and exhaust gases (hydrocarbons, CO, NO_x_). A direct effect of nanoparticles after or upon the translocation of the nanoparticles in DE may play a role, but no direct evidence is presented for this explanation. It is known that upon inhalation a large fraction of nanoparticles will deposit in the nasal cavity [14]. From there DEP, but also carrier constituents such as polycyclic aromatic hydrocarbons (PAHs) and redox metals in DE may migrate through the epithelium to the olfactory bulb [5,6,8] and cause mild inflammation and toxicity[7,8]. Uptake of MnO2 nanopartciles in the olfactory bulb of rats has been reported beyond 6 hours hours after exposure, although no earlier time or inflammatory markers were investigated [7,8]. Therefore we think it is unlikely that the effectis induced by a toxic or inflammatory effect of diesel particles in the brain. In addition, gases such as CO, NO_x _and hydrocarbons may have mediated changes in EEG through vagal reflexes in the airways. Endogenous carbon monoxide (CO) generated by the heme oxygenase system has been shown innumerous studies to play a role in cardiac and neurophysiologic responses, but little information is available whether these findings are also relevant in traffic or diesel exhaust exposure. Interestingly, diesel particles may activate a pro-inflammatory vaso-vagal reflex in rats which is reducedby atropine [15]. This suggests that diesel particles themselves stimulatevagal reflexes in the airways and may as such cause feedback to the brain. On the other hand no difference in heart rate was seen between exposures, which would have occurred of the current diesel exhaust had induced a vagal reflex.

A further complication is that the exposure room was contaminated with a pungent smell and it cannot be excluded that the smell of diesel exhaust plays a role in the increased cortical arousal in the exposed subjects. However a confounding effect on our findings by the smell is unlikely for several reasons. First, subjects reported only discomfort due to smell in the first minutes while changes in EEG were only seen at the end of the exposure interval. Secondly, the effects on MPF continued to rise after subjectsleft the exposure chamber. Thirdly, the diesel odor was also present during the sham condition, because of alternating use of the exposure chamber for diesel and filtered air exposure. Finally, olfactory sensitization to chemical stimulation is usually noted as an increase of slow-wave activity within a few minutes of onset of exposure [16]. This slow wave activity was unaffected during the entire study.

Interpretation of our findings is hampered by the lack of similar studies on such a time-scale. Usually EEG studies are done on short-term exposure or tasks, that last only a few minutes. Therefore we have to consider that the increased activity of the frontal cortex could also relate to lowered vigilance of the subjects, caused by weariness or getting bored during the exposure sessions. Indeed, increased β2 values have been observed in combination with lowered vigilance during cognitive tasks [17] and are often regarded as compensation for lowered vigilance. However this is usually associated with increased levels of EEG slow wave activity [18], which was not observed in this study. In addition, no significant effect of β2 fast-wave activity was seen in the same subjects during sham exposure. Therefore we conclude that decreased vigilance is unlikely to explain the observed effects. Other studies using EEG have linked frontal brain activity to various physiological and psychological findings. Increased β2 levels have been reported in patients with neurological and psycho-pathological disorders, such as headache, post-traumatic stress disorder, burnout and traumatic brain injury, and are often regarded as indicators of cortical stress [19,20]. Although tempting, linking these effects of short-term functional changes in the frontal cortex to decreased cognitive function across assessments of verbal and nonverbal intelligence and memory constructs in epidemiological work [12] is simply not possible at this stage.

Ambient PM exposure has been associated with inflammation in the lung, exaggerated airways responses and increased morbidity as well as mortality inrespiratory and cardiovascular diseases [1,2]. Exposure to diesel exhaust has been associated with similar effects, and has been used as a surrogate exposure in many experimental studies [3,13,15]. The observed effects of diesel exhaust on the EEG add up to the evidence that air pollution may exert its effects by a variety of pathways and may induce effects in the brain. Based on the onset, location, frequency profile and persistence of the response in EEG, we suggest that our findings are due to an effect of nanoparticles that slowly penetrate the brain or affect neurophysiologic signaling. However we can only speculate what these effects on the frontal cortex may mean for chronic exposure to PM and/or nanoparticles. Further studies are necessary to establish whether other nanoparticles induce this effect, and to explore the dose-response relationship and clinical implications of these novel findings. It needs to be realized that further human studies are confronted with serious limitations, since neurochemical work and/or taking samples of spinal fluid are not possible. Subjecting the subjects to non-invasive techniques for imaging (Magnetic Resonance Imaging, MRI) or measuring (Near Infrared Resonance Spectroscopy, NIRS) blood flow and tissue damage are the options that are currently being explored to follow-up these human studies. Current work concentrates on the similar effects ofartificially generated nanoparticles that are not contaminated with gases and other components.

## Authors' contributions

BC and LE carried out the pilot-measurements and data processing of the EEG signals, and prepared and supervised the project on-site; HT, was responsible for the exposure assessment, AB and NM were the responsible medical doctors for the survey; TS and PB were the coordinators and planners of the study. The manuscript was written by BC and PB, but all authors read, corrected and approved the manuscript.
